# Fitness consequences of maternal and grandmaternal effects

**DOI:** 10.1002/ece3.1150

**Published:** 2014-07-19

**Authors:** Roshan Prizak, Thomas H G Ezard, Rebecca B Hoyle

**Affiliations:** 1 Department of Mathematics, Faculty of Engineering and Physical Sciences, University of SurreyGuildford, Surrey, GU2 7XH, UK; 2 Department of Electrical Engineering, Indian Institute of Technology BombayPowai, Mumbai, 400076, India; 3 Centre for Biological Sciences, University of SouthamptonLife Sciences Building 85, Highfield Campus, Southampton, SO17 1BJ, UK; 4 Institute of Science and Technology AustriaKlosterneuburg, Austria

**Keywords:** Adaptation, indirect genetic effect, maternal effect, phenotypic evolution, phenotypic plasticity, quantitative genetics

## Abstract

Transgenerational effects are broader than only parental relationships. Despite mounting evidence that multigenerational effects alter phenotypic and life-history traits, our understanding of how they combine to determine fitness is not well developed because of the added complexity necessary to study them. Here, we derive a quantitative genetic model of adaptation to an extraordinary new environment by an additive genetic component, phenotypic plasticity, maternal and grandmaternal effects. We show how, at equilibrium, negative maternal and negative grandmaternal effects maximize expected population mean fitness. We define negative transgenerational effects as those that have a negative effect on trait expression in the subsequent generation, that is, they slow, or potentially reverse, the expected evolutionary dynamic. When maternal effects are positive, negative grandmaternal effects are preferred. As expected under Mendelian inheritance, the grandmaternal effects have a lower impact on fitness than the maternal effects, but this dual inheritance model predicts a more complex relationship between maternal and grandmaternal effects to constrain phenotypic variance and so maximize expected population mean fitness in the offspring.

## Introduction

Maternal effects occur when maternal phenotype(s) influence offspring phenotype(s) by means other than direct genetic transmission (Mousseau and Fox 1998). This alternative mode of inheritance can act as a divergent or stabilizing force (Räsänen and Kruuk 2007; McGlothlin and Galloway 2014) by, respectively, accelerating adaptation to novel environments (Lande and Price 1989) or by reducing phenotypic variance to maximize population mean fitness in relatively stable environments (Hoyle and Ezard 2012). When the environment experienced by the parent covaries with the environment encountered by the offspring, maternal effects are particularly likely to evolve (Uller 2008; Fischer et al. 2011; Kuijper and Johnstone 2013). Transgenerational effects are not restricted to the maternal generation, however. Phenotypic “memory”, often provoked by environmental factors, can persist for many successive generations (Molinier et al. 2006) and may differ depending on the sex of the influencing ancestor (Lock 2012). In particular, levels of grandmaternal nutrition can, over and above maternal effects, alter life-history traits in the collembolan *Folsomia candida* (Hafer et al. 2011), obesity in humans (Cropley et al. 2006), and mass in white-tailed deer *Odocoileus virginianus* (Mech et al. 1991). Analogously, a grandfather effect was reported for offspring weight in the western mosquitofish *Gambusia affinis* (Reznick 1981). The food environment in the great-grandmaternal generation in soil mites *Sancassania berlesei* can leave a phenotypic signature on life-history traits including egg length (Plaistow et al. 2006). While the potential for multigenerational transgenerational effects on life-history and phenotypic traits is therefore clear, it is not known how these effects from different generations should combine to maximize population mean fitness in the offspring generation. Here, we extend recent modeling work (Lande 2009; Hoyle and Ezard 2012) to find out.

We assess fitness within a focal generation, whose phenotypes are constructed from multiple components. The transgenerational maternal and grandmaternal components assume that the phenotypes in previous generations determine part of the current phenotype under selection and so the fitness belongs (Wolf and Wade 2001) to the current generation. Wolf and Wade (2009) defined a “true” maternal effect as one with a causal link between maternal genotype or phenotype and the offspring phenotype. Within this definition, various subcategories have emerged: Transgenerational effects that enhance fitness have been termed “adaptive maternal effects” (Marshall and Uller 2007), while Räsänen and Kruuk (2007) defined positive maternal effects as ones that (could) accelerate microevolution, presumably toward some shifting optimum. The challenge with more specific terms is that their combinations are not fixed: Hoyle and Ezard (2012) showed how a negative maternal effect is “adaptive” in the sense of Marshall and Uller (2007) in a relatively stable environment, but a positive maternal effect is “adaptive” during adaptation following a rapid shift in environment. We therefore do not assume that a particular maternal effect is adaptive *per se* because this definition requires an understanding of the context in which the organism lives (Plaistow et al. 2006; Plaistow and Benton 2009). Our goal is a better understanding of how positive and negative maternal and grandmaternal effects result in changes to expected mean fitness in the offspring.

The basic framework is a quantitative genetic model of adaptation via plasticity, genetic assimilation (Lande 2009), maternal (Kirkpatrick and Lande 1989; Hoyle and Ezard 2012), and grandmaternal effects. Lande (2009) used this approach to study how phenotypic plasticity and additive genetic variance interact during adaptation to an extraordinary new environment. Hoyle and Ezard (2012) blended this model with that of Kirkpatrick and Lande (1989) to include a maternal effect coefficient *m* that characterized the direct path from maternal phenotype to offspring phenotype, separately from the genetic contribution. Note that this maternal effect is therefore an indirect genetic effect because the maternal phenotype is, in part, genetically determined and the inheritance pathway is not via direct genetic transmission (Moore et al. 1997; McGlothlin and Brodie 2009). We extend the model in Hoyle and Ezard (2012) to include a linear contribution of the grandmaternal phenotype, captured via an analogous (indirect genetic) grandmaternal effect coefficient *g*. Note that we model an explicit grandmaternal effect, as demonstrated experimentally (Hafer et al. 2011; Lock 2012), and not the cumulative consequences of successive maternal effects. We investigate how *m* and *g* combine to determine fitness in two environmental scenarios: at equilibrium and in the immediate aftermath of a sudden environmental shift, because a traditional perspective of maternal effects is that they facilitate adaptation to a changing environment (Räsänen and Kruuk 2007; Uller 2008).

## Materials and Methods

**Figure 1:**
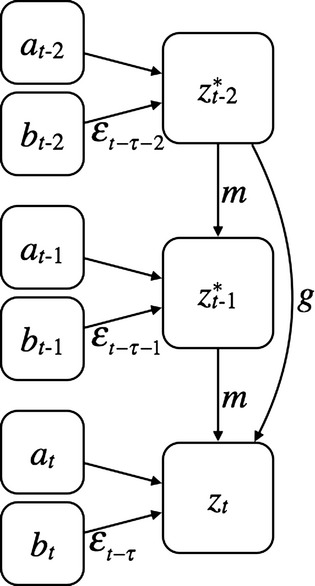
Schematic of model structure showing how 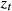, the offspring phenotype in generation *t*, is constructed from additive genetic *a*_*t*_ and phenotypically plastic *b*_*t*_ components. The transgenerational effects are modeled using the maternal coefficient *m* and an analogous grandmaternal coefficient *g*, which link the maternal and grandmaternal phenotypes after selection (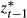 and 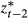, respectively) to 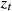. *ε*_*t*−*τ*_ is the environment before selection.

We take a quantitative genetic approach. The model is univariate, asking how a trait affects the same trait in future generations (e.g., Falconer 1965). Generations are discrete and nonoverlapping. We assume phenotypes determine fitness and are constructed from multiple components. The phenotype of an individual at time (≡generation) *t* is:

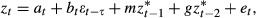
(1)where *a*_*t*_ is the additive genetic component (breeding value) of the phenotype in the reference environment (*ε* = 0) and *b*_*t*_ is the linear slope (reaction norm) of the plastic phenotypic response to the environment *ε*_*t*_ (Lande 2009). We note that the mean breeding value is, by definition, constrained to have a mean of zero. There is a lag *τ*, measured in fractions of a generation, between juvenile development and the time when selection occurs. This lag is a key parameter in disentangling within-generation phenotypic and transgenerational plasticity (Uller 2008; Hoyle and Ezard 2012). The maternal coefficient *m* and the grandmaternal coefficient *g* characterize the influence of the maternal and grandmaternal phenotypes after selection (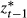 and 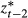, respectively), on the offspring phenotype. *e*_*t*_ is the residual component of phenotypic variation, which we assume to have mean zero without loss of generality. Fig. 1 is a schematic of model structure.

From equation 1, the variance of 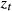 in a constant environment *ε* satisfies:

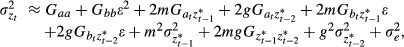
where *G*_*aa*_ and *G*_*bb*_ are the variances of *a*_*t*_ and *b*_*t*_, respectively, and are assumed to be constant. 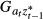 is the covariance of *a*_*t*_ with 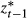; all other covariances are defined in similar fashion. Following Lande (2009), the phenotypic variance is minimized in the reference environment *ε* = 0, which forces the covariance between additive genetic effects and phenotypic plasticity *G*_*ab*_ to be zero.

Also, from equation 1, we see that



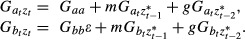


The mean phenotype (an overbar denotes the expectation value) is given by

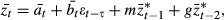


Finally we use

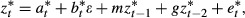
where * denotes values after selection in generation *t*, to see that

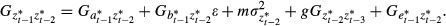


Now, we look for an equilibrium under weak selection, such that:

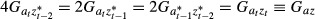


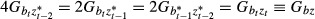


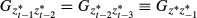


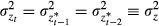


We assume that at equilibrium, under weak selection, the distribution of 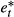 is the same as that of *e*_*t*_ and hence 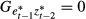.

We find

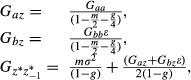
and so

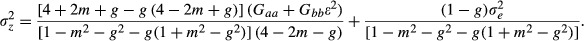


The optimum phenotype is assumed to be a linear function of the environment: *θ*_*t*_ = *A* + *Bε*_*t*_ where *A* and *B* are constants and *ε*_*t*_ is the environment at time *t*. The fitness is defined as a Gaussian function given by

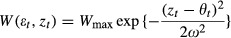
where *W*_max_ and *ω* are constants. Here, *ω*^2^ is the strength of stabilizing selection (width of the fitness function).

Assuming that the phenotype 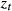 follows a Gaussian distribution, the population mean fitness is given by

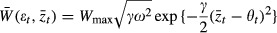
where 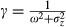.

Following Lande (1979), and using 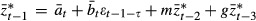 (which assumes no fertility selection), we therefore have

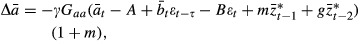
(2)

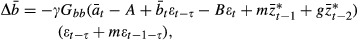
(3)

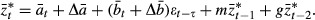
(4)

### Adaptation to new environment

We consider −0.6 ≤ *m* ≤ 0.6 and −0.3 ≤ *g* ≤ 0.3 at increments of 0.05 to investigate how *m* and *g* interact to determine fitness after a sudden environmental shift, modeled as a noisy step change *ε*_*t*_ = *U*_*t*_*δ* + *ξ*_*t*_ (Lande 2009). In this model of environmental change, *U*_*t*_ is the unit step function that jumps from 0 to 1 at *t* = 0, *δ* is the size of the sudden change in average environment, and *ξ*_*t*_ is a Gaussian stationary autocorrelated random process with mean zero, variance 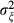, and autocorrelation *ρ*_*τ*_ over the interval *τ*.

Taking the expectation of equations (2–4) over the Gaussian distribution of the stochastic component in the environment *ξ*_*t*_, we have

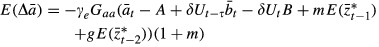
(5)

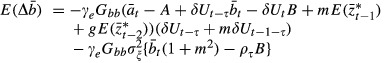
(6)

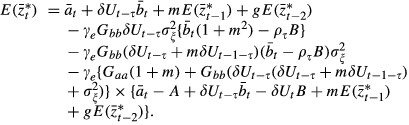
(7)

To get the above equations, we assumed that the stochastic component of the environment is uncorrelated across more than a single generation. We have also approximated *γ* by 
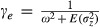
, and, under weak selection, approximated the expected phenotypic variance 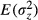 as:

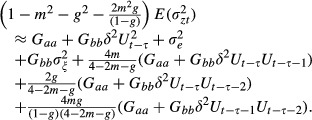
(8)

Note that this is slightly different to Hoyle and Ezard (2012), in that we now incorporate distinct environments after juvenile development (but before selection) in the present, maternal and grandmaternal generations rather than just the environment in the present generation. We expect these to be good approximations for 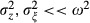. The expected value of fitness is

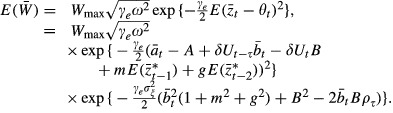
(9)

Commented MATLAB simulation routines are available as online supplementary material.

## Results

**Figure 2:**
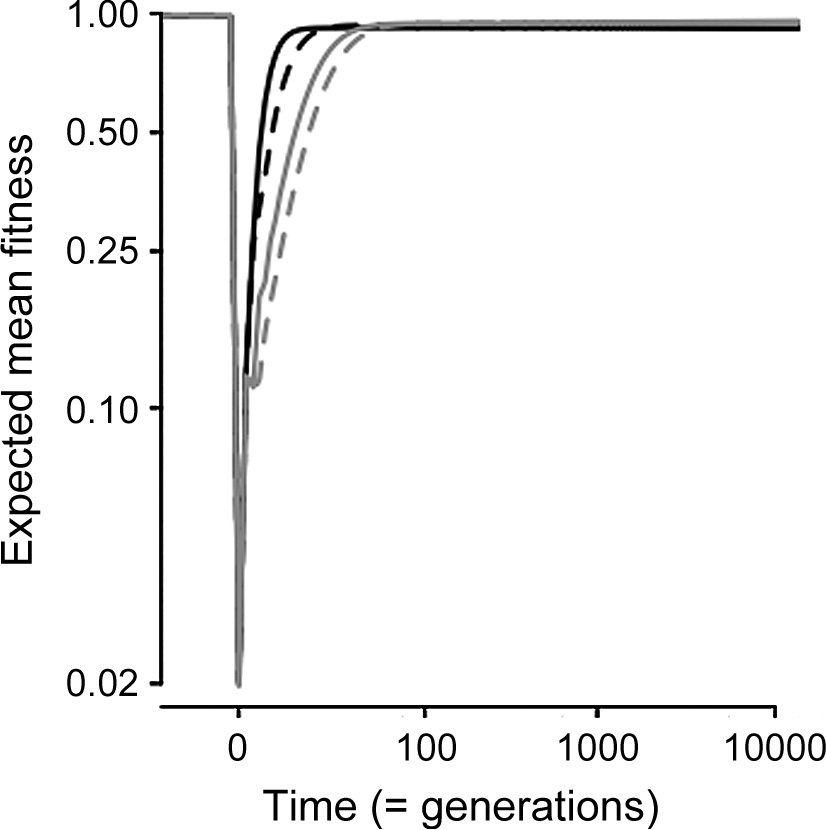
Expected mean fitness (as a proportion of *W*_max_ = 1) through time, plotted on the scale of the natural logarithm. All four combinations of *m* ± 0.35 and *g* ± 0.15 are depicted to illustrate how transgenerational effects combine during adaptation to an extraordinary new environment. Black if *m* > 0 and gray if *m* < 0; solid lines if *g* > 0 and dashed lines if *g* < 0. Positive transgenerational effects lower expected mean fitness (Hoyle and Ezard 2012). Parameter values follow Lande (2009) and Hoyle and Ezard (2012): *A* = 0, *B* = 2, *ρ*_*τ*_ = 0.25, *σ*_*ξ*_ = 2, *G*_*aa*_ = 0.5, *G*_*bb*_ = 0.045, *γ* = 0.02, *ω*^2^ = 50, and *δ* = 10.

**Figure 3:**
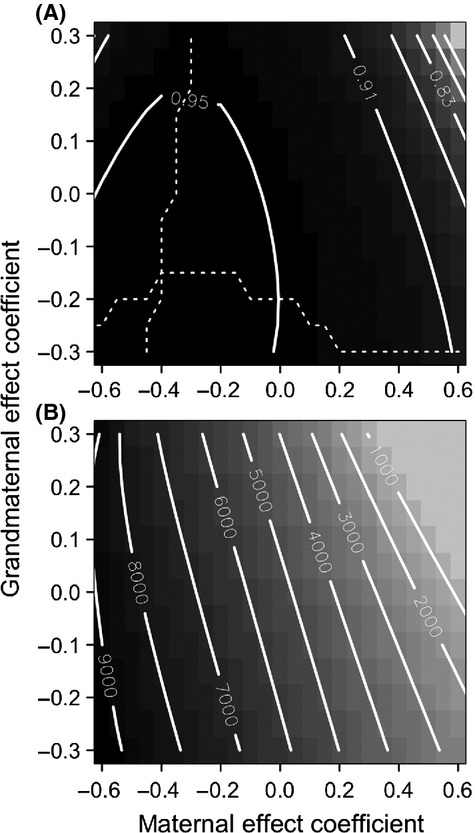
(A) Expected mean fitness (on the scale of the natural logarithm) after 100,000 generations and (B) number of generations to recover equilibrium fitness after the step change in the environment (within 0.0001; see also Fig. 2). The dotted lines in panel (A) indicate maximum expected mean fitness holding either maternal or grandmaternal effects constant. When *g* = 0, the model reduces to the results of Hoyle and Ezard (2012). Parameter values as in Fig. 2.

Multigenerational transgenerational effects change the dynamics during both transient and equilibrium phases (Fig. 2). The potential for maternal and grandmaternal effects to accelerate adaptation to the extraordinary new environment is clear: Adaptation is fastest when *m* > 0 and *g* > 0 and slowest when *m* < 0 and *g* < 0, with *m* being more influential than *g* (Figs. 3). As an example, it takes 5270 generations for expected mean fitness 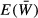 to be within 0.001 of the equilibrium in the novel environment when *m* = 0 and *g* = 0. If *m* = 0.05 and *g* = 0, then this point is reached after 4928 generations. If *m* = 0 and *g* = 0.05, then this point is reached after 5088 generations. 100,000 generations after the step change, expected mean fitness 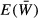 is maximized for *m* = −0.4 and *g* = −0.15 (Fig. 3A). Although *m* > 0 and *g* > 0 accelerate adaptation during the transient phase (Figs. 3), it realizes lower 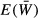 once the new equilibrium is reached. At the new equilibrium (after the step change), *m* < 0 and *g* < 0 maximize 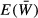. When either *m* or *g* is positive, however, the optimal *g* or *m* (respectively) to maximize 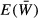 (dotted lines in Fig. 3A) is of the opposite sign.

**Figure 4:**
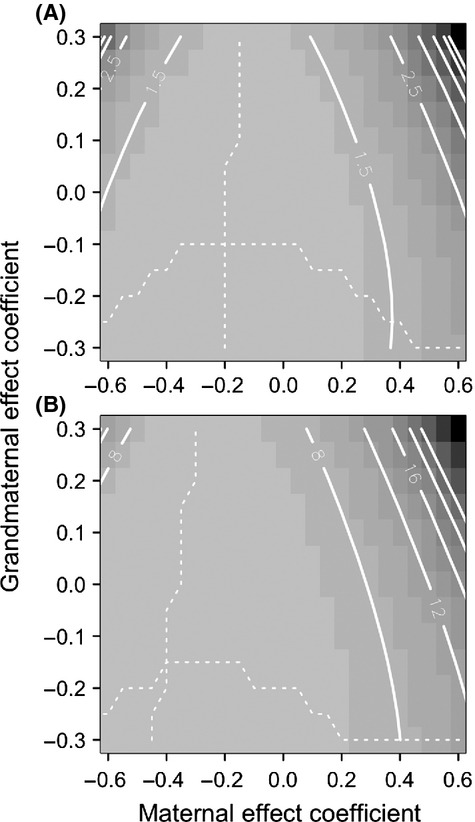
Expected phenotypic variance in the reference (A, *ε* = 0) and novel environment (B, *ε* = 10). The dotted lines indicate minimum expected phenotypic variance holding either maternal or grandmaternal effects constant. Parameter values as in Fig. 2.

The shape of the plateau on the fitness landscape (Fig. 3A) suggests two key points to explain these results: (1) 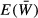 is more sensitive to the phenotype in the maternal generation than the grandmaternal one, particularly if *m* > 0 (compare the distribution of contours in Fig. 3A) and (2) the fitness is largely driven by the combination of multigenerational components that minimize the expected phenotypic variance 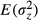 (compare Figs. 3A and 4). The variance in the reference environment is lower than in the novel environment, but the combinations of *m* and *g* that influence it are qualitatively similar (Fig. 4).

## Discussion

This is the first study to investigate how multigenerational indirect genetic effects combine to determine expected mean fitness. We do this using a straightforward conceptual extension of recent theoretical quantitative genetic models (Lande 2009; Hoyle and Ezard 2012). Hoyle and Ezard (2012) showed that negative maternal effects maximize population mean fitness in relatively stable environments because *m* < 0 minimizes phenotypic variance, which keeps more of the population closer to the target phenotype. On an individual level, if *m* is negative, the effect of the maternal phenotype is discounted against the inherited genes and so the phenotype is brought closer to the optimum. The straightforward extension of this logic to multiple generations here realizes nontrivial mathematical expressions (equations 7 & 8), suggesting that the maternal *m* and grandmaternal *g* exert complex effects on the expected phenotypic variance and therefore on phenotypic evolution. In the immediate aftermath of a sudden environmental shift, adaptation to the new optimum is fastest when both *m* and *g* are positive (Figs. 3). Once the new equilibrium is reached, both *m* and *g* should be negative to maximize expected mean fitness 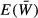 (Fig. 3A).

Experimental work is increasingly documenting how grandmaternal effects alter life-history (Hafer et al. 2011; Lock 2012) or phenotypic (Cropley et al. 2006) traits over and above maternal effects, but, to our knowledge, experiments assessing the fitness implications after controlling for multigenerational factors have not yet been performed. As expected under Mendelian inheritance (Galton 1897), 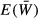 appears more sensitive to changes in *m* than *g* during both the transient and new equilibrium phases. This sensitivity is more complex than a half contribution from mothers and a quarter contribution from grandmothers, however (equation 8). In particular, at equilibrium, the contributions of the two generations are not uniform across all explored combinations of *m* and *g*: Note the vertical and horizontal spread of contours in Fig. 3A. This result is largely driven by the combinations of *m* and *g* that minimize the phenotypic variance in relatively stable environments (Fig. 4). In this regard, the grandparental effects operate in a parameter space determined by the same processes as the parental effects and therefore feed into the same variance minimizing process. When the parental effects are positive, the system then favors antagonistic, even more negative grandparental effects to provide the counterbalance and constrain the phenotypic variance.

For simplicity, we assume that both *m* and *g* are constant and not context dependent (Rossiter 1996; Plaistow and Benton 2009). For example, Plaistow et al. (2006) showed how the persistence (and effect size) of great-grandmaternal, grandmaternal, and maternal effects differed between high- and low-food environments in the soil mite *Sancassania berlesei*. Although work is ongoing to study evolvable context dependence, it will, given the complexity of the expressions derived herein assuming fixed *m* and *g* (equations 7 & 8), prove particularly challenging to incorporate multigenerational effects in this framework. We nevertheless built on the existing quantitative genetic architecture because it neatly incorporates within- and transgenerational plasticity. Understanding how organisms flexibly adjust their phenotypes to match their environment is best achieved through concurrent investigation of both within-generational phenotypic plasticity and transgenerational plasticity via maternal and multigenerational effects (Uller 2008; Ezard et al. 2014). Although our interest here is how maternal and grandmaternal phenotypes influence the same phenotype in the focal generation (e.g., Falconer 1965, for the case of maternal effects only), the overarching concept that transgenerational effects alter phenotypic variances extends into multiple dimensions (Townley and Ezard 2013; Kuijper et al. 2014). The role of dynamic phenotypic variance is key to understanding the multigenerational contributions of mothers and grandmothers to phenotypic evolution (Figs. 4).

## Supporting Information

**Data S1.** MATLAB code to run simulations.
